# Human brucellosis: Widespread information deficiency hinders an understanding of global disease frequency

**DOI:** 10.1371/journal.pntd.0010404

**Published:** 2022-05-17

**Authors:** Christopher G. Laine, Harvey M. Scott, Angela M. Arenas-Gamboa

**Affiliations:** Department of Veterinary Pathobiology, College of Veterinary Medicine and Biomedical Sciences, Texas A&M University, Texas, United States of America; Baylor College of Medicine, UNITED STATES

## Abstract

**Background:**

For decades, human brucellosis has been recognized worldwide as a significant cause of morbidity, yet the annual incidence of this disease remains unknown. We analyzed this frequency, using international reports (2005–2019), identifying information gaps, and distinguishing a possible path forward.

**Methodology/Principal findings:**

A novel approach to estimating the incidence of this disease was explored. We utilized annual health data extracted from the World Organization for Animal Health (OIE)–World Animal Health Information System (WAHIS) database, assessing the dataset completeness and representativeness of the data for the world population. Additionally, we assessed the reported country level human brucellosis case counts and the factors that influenced the observed changes over time. Our analysis revealed incomplete and unrepresentative information, preventing the estimation of annual human brucellosis case incidence at the global level. In the OIE-WAHIS database, only 48.4% of the required reports have been submitted as of 2019, with approximately 47.3% of the world population represented. Additionally, geographic regions were disproportionate in completeness, representativeness, and actual reported case counts. Africa and Asia constituted the majority of reported cases, while simultaneously submitting the lowest percentage of reports as well as covering the lowest percentage of their populations within those reports, when compared to the rest of the world.

**Conclusions/Significance:**

The global annual frequency of human brucellosis cases remains elusive. Furthermore, there exists great heterogeneity in diagnostic, surveillance, and reporting systems worldwide, calling into question the validity of available information. This study reveals that the Neglected Zoonotic Disease priority status for brucellosis should be restored.

## Author summary

Despite brucellosis being a major concern worldwide, particularly for populations residing within resource-limited settings, a suitable estimate of annual incidence is currently nonexistent for human disease. To our knowledge, this is the first study to characterize global human brucellosis frequency by utilizing nationally reported case data supplied to the global intergovernmental public health authorities. The combined records demonstrate that within currently available and established international reporting systems, there are insufficient data to calculate the annual global frequency of human brucellosis. Furthermore, the regional differences in populations represented within reports, as well as actual reported case counts, bias the correct interpretation of the overall human brucellosis disease frequency. This suggests that broadcasting a specific global quantity of new cases each year is misleading and, accordingly, there is presently no way to assess the global public health impact of this disease. In past years, disease prioritization by individual countries, including the reallocation of funds to national surveillance, have been demonstrated to positively impact the completeness and representativeness of the data. In the future, the international community must similarly reallocate resources to understand and fill gaps within the available information. Application of this information can be directed towards effectively pinpointing disease burden and efficient control strategies. The reinstatement of human brucellosis as a priority Neglected Zoonotic Disease by the WHO would substantially facilitate this process.

## Introduction

Brucellosis is a globally distributed bacterial disease that burdens entire populations of livestock and people, as well as their respective economies [1–4]. Three of the *Brucella* species are known to be endemic in most countries [2,4]. These species are highly virulent to both their natural hosts as well as to humans [1,4]. They include *B*. *abortus* that primarily infects cattle, *B*. *melitensis* that infects sheep and goats, and *B*. *suis* which has a tropism for domestic, feral and wild swine populations [4]. Although brucellosis is a disease of considerable importance in livestock, the disease in humans is less recognized, despite the fact that it is associated with substantial and prolonged morbidity [1,4]. Disease in humans is typically characterized by non-specific flu-like illness manifesting as undulating fever, fatigue, sweats, and malaise; coincidentally, akin to the signs and symptoms of malaria, one of the most common infectious diseases of the developing world [1,2,4]. Amongst chronic cases, recurrent fevers, arthritis, myocarditis, and neuropathies can occur [1,4]. Typically, humans are exposed to the pathogen through consumption of unpasteurized milk products and the handling of contaminated tissues, such as aborted placentas [4]. These exposure pathways make raw milk product consumers, animal handlers, abattoir workers, and veterinarians at high risk for acquiring the disease within endemic settings [4]. Additionally, the ease by which *Brucella* species can be transmitted via aerosol imposes a risk of transmission to other lower-risk populations, and has resulted in its classification as a category B bioterrorism agent [5].

Remarkably, despite the longstanding recognition that brucellosis poses a significant threat to both the agricultural and public health sectors around the world [2,6,7], the number of new human brucellosis cases remains unclear. In 2006, a group of researchers attempted to determine the incidence of human brucellosis, concluding that the number of new infections exceeded 500,000 cases annually [8]. Consequently, this number has been repeatedly cited some 632 times over a fifteen-year period [9]. Unfortunately, our investigation into the source of this new case count revealed that this number was not evidence based, as the data and citations within the literature do not support the reported number [8]. Subsequently, two World Health Organization (WHO) commissioned studies published in 2012 attempted to quantify the annual number of new cases via a systematic review and metanalysis of the scientific literature [10,11]. These two articles concluded that it was not possible at the time to accurately determine the global frequency of this disease; largely, because of an insufficient number of studies and a lack of scientifically comparable data [10,11].

Therefore, determination of the distribution and frequency of human brucellosis is vital for truly understanding its impact as well as for developing sustainable control strategies [12]. To the best of our knowledge, the estimation of a global annual incidence of this disease, using data reported by countries to intergovernmental public heath institutions, has not been attempted. Therefore, in this study we aim to: 1) determine if it is possible to estimate an annual global incidence utilizing all available reported data, 2) identify and characterize any knowledge gaps in the available information that hinder such an estimation, and 3) distinguish a possible path forward in the form of an identifiable surveillance initiative made by Kenya’s up-and-coming One Health office; that is, one that could be utilized as a model to understand the disease situation in countries that are not currently providing information about human brucellosis. The findings will provide insight into the necessity for reprioritization of this disease and the redirection of health-related resource allocations.

## Methods

### Evidence before the study

Despite brucellosis being described as a major concern globally, especially for people living in resource-limited settings, a suitable estimate of annual incidence is currently nonexistent for human disease. The last published study that postulated a frequency occurred fifteen years ago [8]; however, that estimate was not evidence based. Since then, new internet-based reporting systems have emerged. Consequently, a current analysis of the newly available evidence is needed. To begin, we searched PubMed on March 15, 2021, using the terms “human brucellosis incidence” (1,271 articles), “global brucellosis incidence” (171 articles), and “global human brucellosis incidence” (142 articles), for scientific articles published between 2006 and 2021. This search revealed two studies: a systematic review of the global burden of human brucellosis, and a systematic review and meta-analysis of disease frequency and severity [10,11]. Unfortunately, these studies concluded that it was not possible to calculate an estimate of human brucellosis incidence at that time based upon the scientific literature due to an insufficient number of studies (n = 29) [10,11]. Furthermore, the studies that were suitable for data analysis only covered 15 countries [10,11].

### Data collection and procedures

Subsequently, in an effort to quantify incidence using intergovernmental health institution data, we approached the WHO, Food and Agriculture Organization of the United Nations (FAO), and United States Centers for Disease Control and Prevention (CDC) in an attempt to gather information on global human brucellosis frequency. Although these institutions did not directly provide information, during our search of its website, the WHO indicated that the World Organization for Animal Health (OIE) had become the primary intergovernmental organization collecting and disseminating human brucellosis knowledge from each Member State [13]. The OIE aggregates and presents disease data via an online database called the World Animal Health Information System (WAHIS) [14]. Established in 2005, the WAHIS aggregates self-reported data from the OIE’s 182 permanent member countries, reflecting information on domestic animals and wildlife, as well as emerging diseases and zoonoses [14]. Every year, the OIE expects an annual report from each of their member countries that includes an update on the number of new human cases of zoonoses [14]. These reports are not mandatory, and the OIE does not confirm that the reported cases are actual cases, or that all of the diagnosed cases in a country are reported.

As the zoonosis section of the OIE annual reports has not yet (December 2021) been integrated into the publicly available WAHIS, we directly contacted the OIE World Animal Health Information and Analysis Department for access to the dataset. Included in the WAHIS information are the year, country name, disease status, and the case count of newly diagnosed human brucellosis (i.e., annual cumulative incidence) provided by each country within their respective annual reports (S1 File) [14]. These reports do not specify which diagnostic tests or clinical criteria were utilized to diagnose or confirm the disease, and do not further specify the *Brucella* spp. infecting humans (e.g., *B*. *abortus*, *B*. *melitensis*, and/or *B*. *suis*) [14], reducing the usefulness of the data for control purposes. Without knowledge of the diagnostic techniques, as well as the surveillance or reporting systems, we could not confirm that the number of newly diagnosed infections presented within the database was valid. Therefore, we decided to refer to the reported numbers as “reported case counts” (RCC). After acquiring the dataset, to ensure best reporting practices for the data, we conducted the rest of our investigation under the Guidelines for Accurate and Transparent Health Estimates Reporting (GATHER) [15].

To begin the analysis, the entire dataset was retained and stored. Although there is a visible drop in the amount of information contained within the dataset for 2019, presumably due to reporting occurring in 2020 amidst the COVID-19 outbreak, we did not disqualify it from inclusion. The raw information supplied by OIE WAHIS is presented in S1 File. Subsequently, to provide a means of comparing data within the dataset we scaled and categorized the information in three different ways. First, to provide a scale comparison of reports, we added national populations from the United Nations (UN) population estimates for each year (2005–2019) [16,17], into the dataset (S2 File). Second, to provide a means of geographical comparison, each of the individual OIE member countries was grouped into one of five continental regions (Africa, Americas, Asia, Europe, or Oceania) as specified by the OIE (S2 File) [14]. Third, for an investigation into the different ways that human brucellosis cases were reported, we categorized each report into a mutually exclusive group (informative versus uninformative) based upon how the information was presented. Informative reports specified a quantified RCC within the report (RCC ≥ 0). Uninformative reports did not provide any quantified information on the brucellosis status of the country.

Following scaling and categorizing, the dataset was assessed for completeness and its representativeness of the world population. To investigate the completeness, we calculated the percentage of informative reports. We then assessed the differences between regions, as well as changes over time. To investigate the representativeness of the world population, we assessed the percentage of the population represented within each region. We charted the percentage of regional populations that were represented by the informative reports annually, demonstrating on average which populations were primarily being represented over time.

Subsequently, we assessed the existing data (i.e., RCCs), analyzing the distribution of reported disease. For this, the actual number of reported cases of human brucellosis was charted by region, displaying the differences in the magnitude of regional RCCs over time. Furthermore, we investigated factors that could influence the change in RCCs over time. We investigated an association between the number of countries providing RCCs each year and the annual total RCCs, providing an indication of the effect that report provision has on RCCs. Additionally, after identifying a sudden drastic increase in RCCs, we investigated the effect that a single country that suddenly prioritizes human brucellosis surveillance can have on the overall interpretation of regional and global data.

### Statistical analysis

Statistical and graphical analysis was conducted using GraphPad Prism 9. To evaluate the disproportionate completeness of informed reporting and population representation, paired sample t-tests were performed to assess the difference in the percentage of countries providing a RCC within each region, as well as the percentage of their populations that are represented within those reports, from the associated worldwide mean (μ). Using simple linear regression, trend lines were fitted for these two variables against the time (reporting year) variable, as well as the actual regional and global RCCs. These trends were then assessed for significance in the slope to investigate change. Finally, the Pearson product-moment correlation coefficient (*r*) and coefficient of determination (*R*^2^) were calculated to assess the relationship between the number of countries submitting informed reports and the total RCC. Uncertainty is represented by standard deviation (σ), while confidence was assessed at 95% and statistical significance at p < 0.05.

## Results

A review of international public health organization (WHO, FAO, and CDC) surveillance datasets revealed that the OIE’s WAHIS database constitutes the only available international aggregate of human brucellosis cases (as of 2021). Of the 193 countries recognized by the UN, 182 are permanent members of the OIE and each of these are expected to submit an annual report that includes information on the occurrence of zoonotic diseases. It is the expectation that all documented cases of human brucellosis are included within these reports [14].

**Fig 1:**
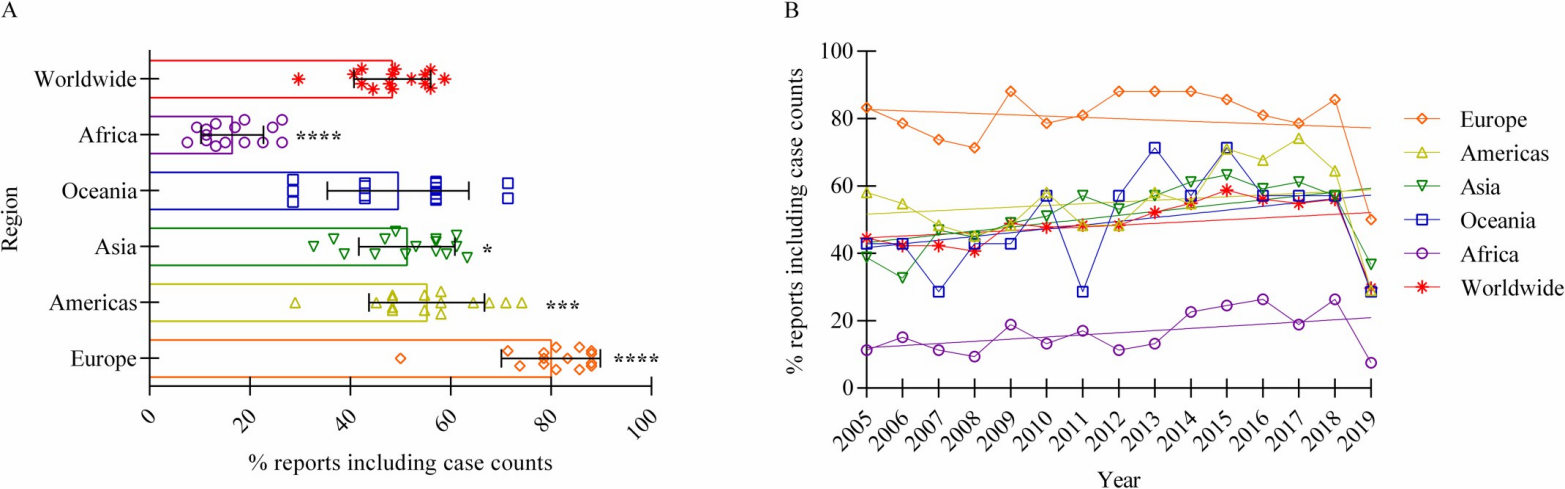
Percentage of OIE annual reports providing case counts of human brucellosis (informed reports), by region (2005–2019). A: Percentage of reports submitted to the OIE classified as informative, separated by region and compared to the entire world (2005–2019). Each point on the plot denotes the percentage of reports within a region that included a case count of human brucellosis during a single year. Bars signify mean and standard deviation. Statistical significance compared to the worldwide mean are denoted by asterisks (* through **** → low through high significance). p-value for Africa is < 0.0001, the Americas = 0.0009, Asia = 0.0363, Europe < 0.0001, and Oceania = 0.6566. B: Proportion of reports submitted to the OIE classified as informed, worldwide and separated by region, plotted over time (2005–2019). Linear regression indicates that the Asian region has the only significant change with an increase (p = 0.038). Worldwide, African, American, European, and Oceanian regression lines are all stable with slopes not significantly diverting from zero (p = 0.26; 0.086; 0.47; 0.53; 0.20 respectively).

Our initial analysis of the data provided by the OIE (S1 File), demonstrates that global human brucellosis RCCs have been incomplete over time due to the lack of comprehensive reporting [14]. Between 2005 and 2019, a total of 2,730 (182 member states multiplied by 15 years) RCCs (i.e., informative reports) were expected to have been received by the OIE, yet only 48.4% (1,322 / 2,730) (σ = 7.7%) were provided. This gap in information represents a large amount of the uncertainty that hinders an accurate quantified annual disease frequency. After identifying the low percentage of reports providing RCCs, the informative reports were assessed to determine their origins. Fig 1A illustrates these reports separated into their respective geographic regions, demonstrating differences in reporting comprehensiveness, with Africa submitting the lowest percentage of reports (μ = 16.5%; σ = 6.2%) and Europe the highest (μ = 80.0%; σ = 9.8%). Fig 1B illustrates that Asia developed the only significant change in the number of informed reports submitted with an increase (p = 0.038), leaving the worldwide and the other regional trends stable with slopes not significantly different from zero. This indicates that the data are not representative of the entire population as there is an information imbalance between geographic regions.

**Fig 2:**
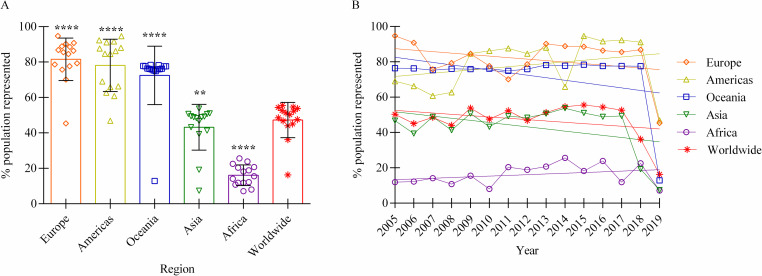
Percentage of populations represented by OIE-WAHIS reports on human brucellosis (informative reports), by region (2005–2019). A: Proportion of each region’s population that are represented within the informed reports, compared to the world as a whole (2005–2019). Each point on the plot denotes the percentage of a regional population that is represented during a single year. Bars signify mean and standard deviation, and level of significance when deviating from the worldwide mean are characterized by asterisks (* through **** → low through high significance). p-value for Africa < 0.0001, the Americas < 0.0001, Asia = 0.0031, Europe < 0.0001, and Oceania < 0.0001. B: Proportion of each region’s population that are represented within informed reports, plotted over time (2005–2019). Linear regression indicates that there was no significant change in representation over time whether worldwide (p = 0.22), Africa (p = 0.26), Asia (p = 0.12), the Americas (p = 0.32), Europe (p = 0.25), or Oceania (p = 0.15).

In light of the significant differences in reporting comprehensiveness between regions, the possibility of population underrepresentation within reports was then further explored. This is important because each country submits one report per year, but not every country contains an equal population. Over the 15-year timeframe, only 47.3% of the average worldwide population has been represented (σ = 10.0%) (Fig 2A). Strikingly, when analyzed by region, it is clear that only 16.1% (σ = 5.8%) of the African population is represented by these reports, in contrast to 81.5% (σ = 12.0%) of Europeans (Fig 2A). When we looked closer, this isn’t surprising since 41.5% (22 / 55) of the African nations have never submitted an informative report, compared to 0.0% (0 / 42) of European countries (S2 File). Fig 2B illustrates that there is no significant change in this representation over time, either worldwide or regionally. This demonstrates that there is not only a low overall percentage of the world population represented every year, but a significant difference between the regions and populations being routinely represented. This clearly implies that the available information is not representative of the entire world population.

**Fig 3:**
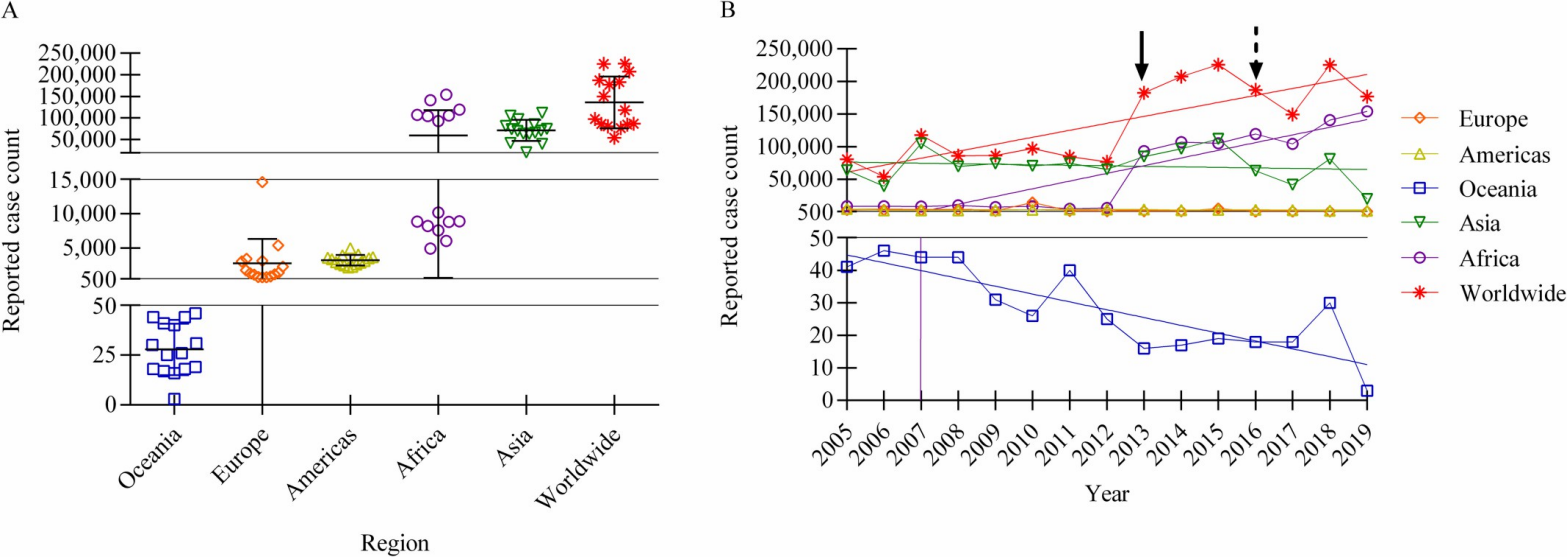
Case counts of human brucellosis reported to the OIE, by region (2005–2019). A: Number of reported case counts (RCC) worldwide and by region (2005–2019). Each point on the scatter plot denotes the actual number of human brucellosis cases reported during a single year. Bars signify mean and standard deviation. B: Number of reported case counts (RCC) worldwide and by region plotted over time (2005–2019). Linear regression indicates that significant change only occurred in the reported case counts worldwide (p = 0.0004), in Africa (p < 0.0001), and in Oceania (p < 0.0001). The solid line arrow signifies the year Kenya began routinely submitting informative reports. The dashed line arrow signifies the year that China discontinued reporting.

After distinguishing that the dataset isn’t adequately complete and isn’t representative of the population, the RCC data can be assessed. When the worldwide total RCCs are analyzed by region, it is evident that Asia (μ = 60.3%; σ = 25.6%) and Africa (μ = 34.0%; σ = 28.5%) encompass the majority of cases over the observed timeframe, while the Americas (μ = 2.96%; σ = 1.65%), Europe (μ = 2.74%; σ = 3.74%), and Oceania (μ = 0.03%; σ = 0.02%) have had a relatively low impact on the total (S2 File). The disproportion can be further appreciated in Fig 3A, which plots the actual RCCs worldwide and by region. Furthermore, when the RCCs are plotted over time (Fig 3B), significant increases in reported cases are observed worldwide (p = 0.0004). Breaking this down by region, significant changes in the number of RCCs were evident in Africa and Oceania, with Africa increasing (p < 0.0001) and Oceania decreasing (p < 0.0001) over time (Fig 3B). These data further demonstrate the critical differences between regions and indicates, counterintuitively, that the cases are heavily weighted towards regions, such as Africa; albeit, with less representation.

## Discussion

This study provides an account of the human brucellosis cases being reported worldwide, clearly revealing a disturbing reality. It is believed that brucellosis in animals imposes a substantial burden on humans in many countries around the world [2]; but, despite being one of the oldest recognized diseases, the number of human annual cases is still unknown. Our analysis revealed that there is only one database aggregating the reported country level case counts (RCCs), and the information within this dataset is both incomplete and unrepresentative of the worldwide population. We also discovered that the majority of the reported cases of human brucellosis come from the regions that provide the least number of informative reports. These two factors, coupled with the continuing increase in the world population, especially in Africa (from 2005 to 2019 Africa’s population increased some 42.84%, shifting the balance of the global population) (Table 1), means we actually have less information now than we did in the past. Unfortunately, extrapolating the data to the other countries within the regions that underreport is not appropriate, due to the amount of data missing from these regions. Furthermore, extrapolation to regions that underreport isn’t acceptable due to heterogenous disease ecologies among regions that reduce data generalizability. This indicates that despite the good intention of scientists to give an annual global frequency in the past, it has clearly not been scientifically correct to do so. Therefore, something must be done to remedy the knowledge gap.

**Table 1:** Regional demographics (2005–2019).

	Average population (in billions)	Average % of world population	Change in population (in billions)	Change in % of world population	% change in population
**World**	7.09	100.0%	1.16	0.00%	17.87%
**Asia**	4.26	60.2%	0.62	-1.16%	15.63%
**Africa**	1.10	15.5%	0.39	2.98%	42.84%
**Americas**	0.95	13.4%	0.13	-0.37%	14.62%
**Europe**	0.74	10.4%	0.02	-1.48%	2.26%
**Oceania**	0.04	0.5%	0.01	0.03%	25.63%

Worldwide population distribution and its growth, represented by 1) Average world population divided amongst the regions, 2) Average proportion of the world population each region holds, 3) Change in the actual population, 4) Change in the average proportion of the world population each region holds, and 5) Percentage change in the population.

**Fig 4:**
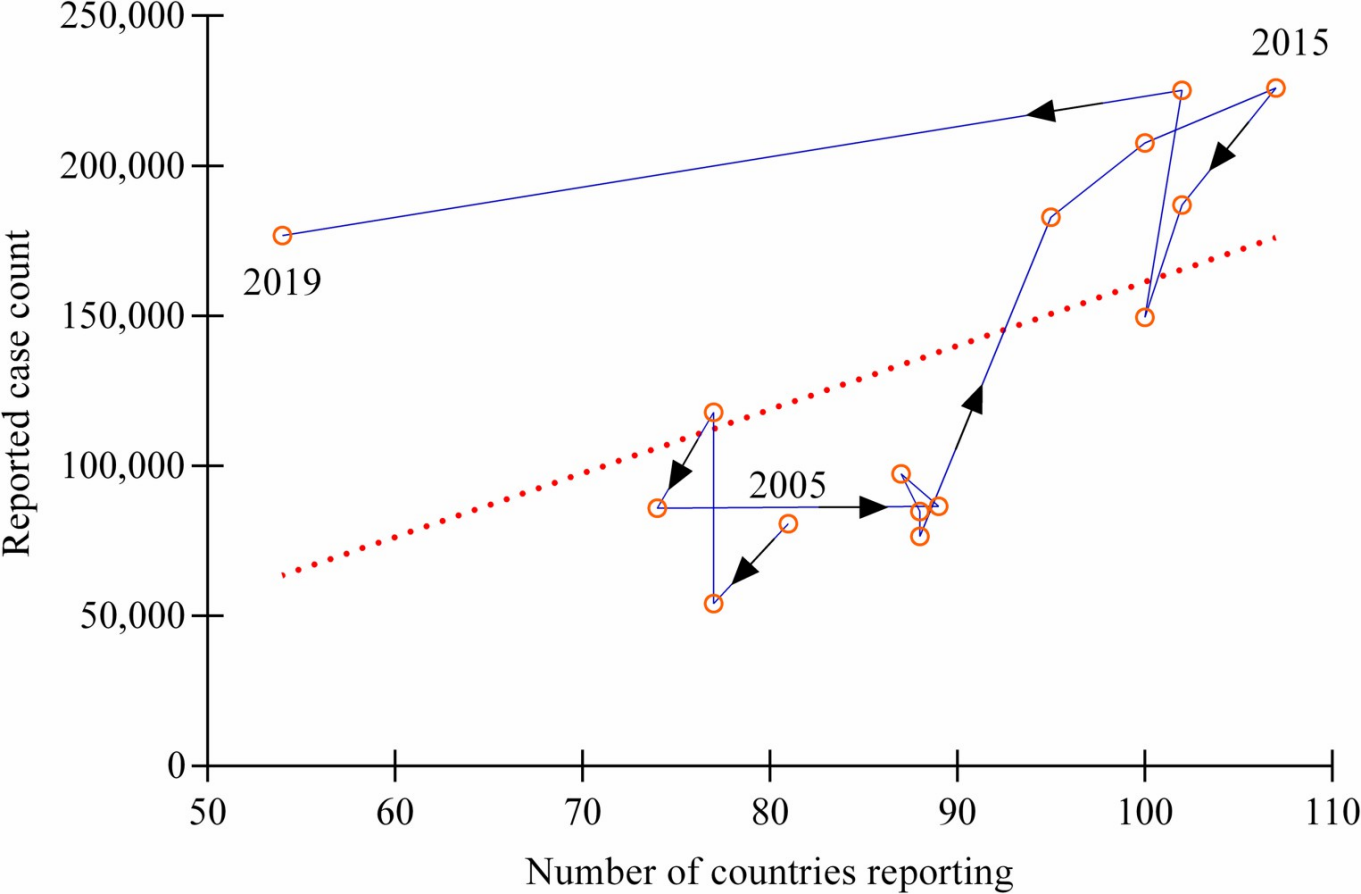
Association between reporting and case counts, globally (2005–2019). Reported case counts worldwide are plotted in comparison with the number of countries submitting informed reports. Arrows indicate progression of time. The plot was then analyzed for correlation (*r* = 0.49) and fitted with a line of best fit which isn’t statistically significant (p = 0.061). This indicates the number of countries submitting informed reports doesn’t significantly influence the total number of reported cases worldwide.

Although the dataset isn’t useful for generating an annual global incidence of brucellosis in humans, it does reveal some interesting facts. Interestingly, the RCCs aren’t increasing because there are more overall countries reporting. An analysis into the relationship between the number of reports that include RCCs (informative reports) and the actual RCCs revealed that there is no significant relationship between the two, as the *r* and *R*^2^ don’t significantly deviate from zero (p = 0.061) (Fig 4). This is unsurprising, as the only significant change in reporting was observed within Asia (Fig 2) and suggests that there are other important factors that influence the increase in RCCs besides the change in overall countries reporting.

**Fig 5:**
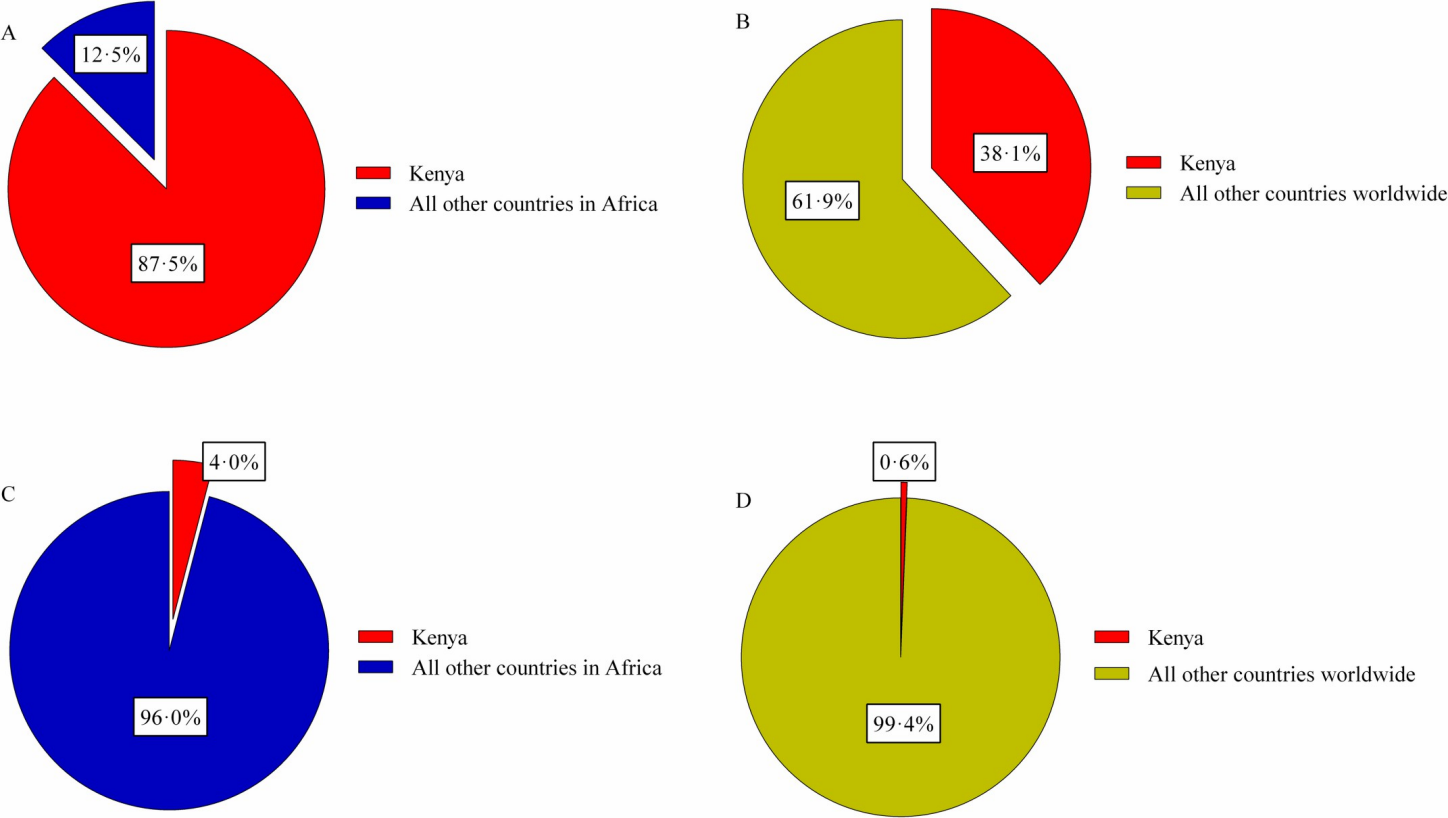
Influence of Kenya on the global and regional reported case counts of human brucellosis (2005–2019). A: Reported case counts compared to the remainder of Africa (2005–2019). B: Reported case counts compared to the remainder of the world (2005–2019). C: Population compared to the remainder of Africa (2005–2019). D: Population compared to the remainder of the world (2005–2019).

Important for the change in total RCCs are countries like Kenya, which between 2005 and 2019 represented 87.5% (776,146 / 887,504) (σ = 46.7%) of all RCCs in Africa (Fig 5A) and 38.1% (776,146 / 2,038,538) (σ = 31.3%) worldwide (Fig 5B). Kenya only began routinely submitting informative reports in 2013 (S1 File). This was shortly after the country established a One Health office and prioritized brucellosis as one of the top ranked zoonoses for surveillance, prevention, and control [18,19]. The magnitude of the Kenyan RCCs is in stark contrast to the country’s population compared to the rest of Africa and the world, averaging 4.0% (σ = 0.02%) (Fig 5C) and 0.6% (σ = 0.04%) (Fig 5D), respectively (2005–2019). This equates to 4.0% of Africa comprising 87.5% of the region’s RCCs, as well as 0.6% of the world comprising 38.1% of the worldwide RCCs (Fig 5). Inexplicably, Kenya annually reports zero cases of the disease in their animal populations [14]. As human disease is directly associated with disease in animals [4], it simply is not plausible that Kenya with its high human RCC has a disease-free animal population, indicating a major discrepancy in reporting systems. The magnitude of the effect that Kenya has on the dataset is important. It represents the degree to which one country investing in surveillance and reporting influences the overall available information.

Conversely, the lack of reporting also significantly biases data interpretation. It is important to note that although Asia holds 26.9% (49 / 182) of the member states, it contains approximately 60.2% (4.26 billion / 7.07 billion) (σ = 0.38%) of the global population (Table 1). Furthermore, China and India’s populations, 1.40 (σ = 0.03) and 1.30 (σ = 0.07) billion, respectively, account for 63.4% (2.70 billion / 4.26 billion) (σ = 0.50) of the Asian population, on average (S2 File). This implies that these two countries (i.e., two annual reports), on average, encompass 38.2% (2.70 billion / 7.07 billion) (σ = 0.53) of the global population each year. The size of these populations allows the potential for any change in reporting from either of these countries to significantly alter the worldwide data for a given year. For example, China’s last report was submitted in 2015 (S1 File), thereafter diminishing the Asian and worldwide RCCs as well as severely decreasing the regional and global populations represented (Fig 2). India only reported twice during the observed timeframe (2016 and 2017) (S1 File), briefly expanding the representation in those years’ RCCs (Fig 2). Consequently, an observed drop in the Asian and global RCCs can be observed in 2016 (Fig 3), but the visual reduction in representation is delayed until 2018 (Fig 2). Unfortunately, it is apparent that the disease is still present in these countries due to the overwhelming number of research studies performed in these locations that reveal a completely different picture [20–25]. This is also true in places like East Africa (other than Kenya) where research indicates human brucellosis is present, but there is no reporting of the disease [26]. With these factors being taken into consideration, it is likely that the RCCs would change drastically if surveillance and reporting systems were capable of gathering and transmitting the complete information.

It is important to understand that when assessing the currently available information, there is low certainty that the RCCs are a true representation of the actual frequency of disease, mainly owing to the surveillance and reporting systems that provide the information. Even in the countries that have RCCs, the data likely do not provide a full picture of the actual disease frequency. This aspect is evident as many individual countries not only report sporadically, but also have great variability in their RCCs (S2 File). It is well known that brucellosis diagnostics and surveillance activities are complex and challenging to achieve, especially in resource-limited settings [12,27–30]. These complexities may be highly significant component causes of worldwide underreporting [12,27–30]. This is attributed to several factors. First, brucellosis can manifest in many different ways and differs across the stages of disease (i.e., acute through chronic, and symptomatic vs. asymptomatic) [1,4]. To complicate the issue, many physicians are unaware of the disease in humans or do not understand proper detection and treatment strategies [31]. Further complications in clinical diagnosis arise due to brucellosis being endemic in most regions in which malaria, typhoid, and other diseases with similar presenting signs are endemic [2]. Consequently, correct diagnosis requires well-trained healthcare professionals to properly differentiate between the causative agents as well as identify co-infections [1,2,22]. For example, one clinical study in Kenya calculated that approximately 13.7% of the acute febrile illness cases within an at-risk zone were positive for brucellosis and that 81.5% of the cases had earlier been misdiagnosed as something other than brucellosis [22]. Second, across the diverse range of tests employed for laboratory diagnostics, each requires distinctively equipped laboratories as well as data analysis capabilities.

Additional computational resources are needed for the purpose of continuous standardization, validation, and calibration, providing the means for effective and efficient identification of disease status. Furthermore, a considerable amount of time as well as sufficient monetary, human, and supply chain resources are also required. To intensify these difficulties, when diagnosing human brucellosis, there are assorted standards of proof in the case definition that differ between organizations. For example, the CDC has a set of criteria for diagnosis that differs from that of the WHO, likely due to the differences in accessibility of diagnostic assays amongst the various nations within the global community [4,32]. Each of the distinctive testing strategies must be interpreted in a specific and unique way, and these are not always comparable across strategies. Furthermore, the only “gold standard” method of identifying a true positive is isolation of the bacterium, which necessitates a substantial amount of biomedical and human resources to conduct [4,27,33]. Additionally, for the purpose of control, it is important to determine the *Brucella* spp. (e.g., *B*. *abortus*, *B*. *melitensis*, and/or *B*. *suis*) that is responsible for human infection in each country and region. This provides evidence as to where to focus interventions (e.g., cattle, goats, sheep, and/or pigs). Unfortunately, identifying the bacterial species requires even more of these expensive resources. Most importantly, resource-limited settings are less likely to be able to support adequate diagnostics. The third challenge is that once there has been a correct positive diagnosis in the laboratory, there must be clear communication and data transfer between laboratory, medical, and public health personnel. Furthermore, proper storage of the data and reporting the findings to the respective authorities is essential. Reporting cases requires a full range of critical infrastructure [12,29,30], while resource-limited settings are less likely to be able to support this infrastructure. Finally, there should be an incentive to report. Without an incentive, many countries will simply allocate their scarce resources elsewhere.

Over time, lacking sufficient empirical evidence regarding the incidence of this disease [10,11], the WHO has withdrawn its priority Neglected Zoonotic Disease status [34]. The importance of this status is nested in the WHO considering these diseases a priority for reduction, owing to the marginalized populations that they impact, and in recognition that the management of these diseases requires an integrated health systems approach and consequently, the reallocation of scarce resources [35]. Kenya is an ideal example of how the prioritization of surveillance, prevention, and control can fill the human brucellosis information gap for a single country and drastically influence regional and worldwide knowledge. Unfortunately, due to the withdrawal of international prioritization, populations afflicted by brucellosis are currently required to fund their own integrated multiphase responses. Furthermore, adequate transportation, education, energy, communications, government facilities, healthcare and public health, information technology, and financial services, as well as continuous interdisciplinary cooperation are fundamental systems necessary for building and sustaining surveillance infrastructure [12,29,30].

Additionally, institutions must have the capacity to provide services to populations that may not be sedentary (e.g., transhumant farmers and nomadic peoples). Most of these populations live in the type of resource-limited settings that lack the basics of a critical infrastructure that are required to conduct any of these activities. To further complicate things, when reporting cases at the international level, information transfer is left to the “honor system”; often, in a setting in which there is a substantial economic disincentive for broadcasting the presence of the disease due to loss of trade in animals and animal products. Ultimately, the absence of formal testing or reporting does not necessarily indicate that the disease isn’t present and transmitting. Therefore, lack of reporting should not be construed as a disease negative status, but rather added evidence of the neglected status of human brucellosis.

The status quo leaves the world in a quandary. Billions of the world’s most vulnerable populations are left to fend for themselves against an insidious disease that in many cases they don’t even know exists [31]. In turn, hundreds of millions are exposed to *Brucella* routinely throughout their everyday lives, while millions of these people most likely contract the disease each year. Therefore, it appears that the only way forward is to prioritize resource allocation towards the infrastructure that facilitates data acquisition, information transparency, and reporting for this truly Neglected Zoonotic Disease.

### Limitations

The primary limitation of this study is that the absence of reliable information foments uncertainty at multiple levels within the analysis. This underlying fact is the primary motive for our conclusion.

## Supporting information

S1 File Raw data from OIE-WAHIS. (XLSX)

S2 File Curated datasets. (XLSX)
